# Human–Animal Relations in Business and Society: Advancing the Feminist Interpretation of Stakeholder Theory

**DOI:** 10.1007/s10551-021-04840-1

**Published:** 2021-05-21

**Authors:** Linda Tallberg, José-Carlos García-Rosell, Minni Haanpää

**Affiliations:** 1 Department of Management and Organisation, Hanken School of Economics, Arkadiagatan 24, 00100 Helsinki, Finland; 2 Faculty of Social Sciences, University of Lapland, Yliopistonkatu 8 A, 96100 Rovaniemi, Finland; 3 Faculty of Social Sciences/MTI, University of Lapland, Yliopistonkatu 8 A, 96100 Rovaniemi, Finland

**Keywords:** Animals, Affective embodiment, Ethnography, Ethics of care, Stakeholder theory

## Abstract

Stakeholder theory has largely been anthropocentric in its focus on human actors and interests, failing to recognise the impact of nonhumans in business and organisations. This leads to an incomplete understanding of organisational contexts that include key relationships with nonhuman animals. In addition, the limited scholarly attention paid to nonhumans as stakeholders has mostly been conceptual to date. Therefore, we develop a stakeholder theory with animals illustrated through two ethnographic case studies: an animal shelter and Nordic husky businesses. We focus our feminist reading of Driscoll and Starik’s (J Bus Ethics 49:55–73, 2004) stakeholder attributes for nonhumans and extend this to include affective salience built on embodied affectivity and knowledge, memories, action and care. Findings reveal that nonhuman animals are important actors in practice, affecting organisational operations through human–animal care relationships. In addition to confirming animals are stakeholders, we further contribute to stakeholder theory by offering ways to better listen to nontraditional actors.

## Introduction

In Zoopolis, Donaldson and Kymlicka (2011) argue that humans and animals are bound in a complex web of relationships which demands a re-evaluation of the status of animals in society. To some extent, animal protection laws and regulations are being reassessed (see Connolly & Cullen, 2018); for example, in 2015, Judge Elena Liberatori ruled in favour of Sandra, a 33-year-old orangutan kept in an Argentinian zoo, granting her legal personhood. The verdict, which found in favour of animals as sentient beings with basic individual rights, was influenced not only by legal aspects but also by the Judge’s affective connection to Sandra (Gonzales, 2019). Further, Pizza, known as “the saddest polar bear in the world”, broke the hearts of millions of people in 2016 as images of her living in a small enclosure in a Chinese shopping mall made headlines worldwide. The emotional public response triggered a series of complaints against both the mall and local authorities, leading to her relocation and the closure of the exhibition. In addition to legal and political aspects, affection and emotions play a key role in shaping public discourse and moral deliberation on the role and treatment of animals in society. The respective cases of Sandra and Pizza arguably signify a small shift in societal anthropocentrism away from considering animals purely as property to considering their feelings and wellbeing beyond human ends. Until recently, the denial and silence of the nonhuman world in organisations created powerful “categorical binaries to reduce other‐than‐male [human] actors to ‘things’ (at worst) and ‘other’ (at best)” (Sayers et al., 2019, p. 239). More interconnected and embodied readings of taken-for-granted classifications and theories may offer new ways of relating, valuing and living in a diversely cohabited world. One such grand theory is stakeholder theory, which has predominantly been reserved for humans. We believe this is due to the heavy influence of the economic and individualistic autonomous-masculine perspective in the field, which a feminist interpretation of stakeholder theory seeks to readdress (cf. Burton & Dunn, 1996; Wicks et al., 1994), facilitating the consideration of nontraditional stakeholders, such as nonhumans in certain contexts.

The question of whether nonhumans can be considered stakeholders has been addressed by several scholars in recent decades (Driscoll & Starik, 2004; Laine, 2010; Starik, 1995; Waddock, 2011). Nevertheless, this work has predominantly revolved around considering the natural environment as a stakeholder of the firm, while animals have received limited attention (see García-Rosell & Tallberg, 2021; Smart, 2021 for exceptions), especially inside organisations. Nonhuman stakeholders remain a controversial issue, perhaps due to most of the work being conceptual and situated within the deontological realm of stakeholder theory. However, current Anthropocene challenges promote the consideration of nonhumans further in business theories. If we agree that the ecological challenges affecting organisations are the result of anthropocentric value-creation models based on profit maximisation (Heikkurinen et al., 2019; Hoffman & Jennings, 2021), then it may not only be short-sighted but also unethical to limit managerial practice and decision-making exclusively to humans. Indeed, the lack of nonhuman inclusivity in stakeholder theory is problematic as it contributes to reifying organisational practices that position humans above other living beings, thus reproducing a worldview in which nonhumans are considered only insofar as they have a direct instrumental value to humans (cf. Heikkurinen et al., 2019; Waddock, 2011).

Considering the conceptual contestability of stakeholder theory, it is not surprising that scholars have been reluctant to accept nonhuman stakeholder status. After three decades of extensive research, there is still a lack of consensus on what constitutes stakeholdership (Miles, 2017). It is not simply that stakeholder scholars are deliberating as to whether nonhumans can be considered stakeholders, but rather that in relation to humans there remain questions about whose interests matter and whose do not count. One theoretical framework used to define stakeholdership is Driscoll and Starik’s (2004) extension of Mitchell et al.’s (1997) stakeholder salience model. According to this framework, stakeholdership is determined through the saliency of power, legitimacy, urgency and proximity (see Driscoll & Starik, 2004 for an overview of these attributes). While Driscoll and Starik (2004) refer to proximity in terms of spatial closeness, Lähdesmäki et al. (2019) conceptually expand proximity to include close emotional bonds and affective social relationships through an ethic-of-care framing. It is from here we develop a stakeholder theory inclusive of nonhumans using a feminist reading of the embodied, relational and affective aspects of the attributes.

This article aims to theoretically advance nonhumans, specifically animals, as stakeholders through our empirical application of feminist care ethics (Connolly & Cullen, 2018; Donovan, 2006) to stakeholder salience (Driscoll & Starik, 2004; Lähdesmäki et al., 2019) in human–animal organisational settings. Our main question is: *How are animals stakeholders?* And secondly: *How are the stakeholder attributes manifested in human–animal care relationships?* Thus, we examine two case contexts involving human–animal relationships: an animal shelter and Nordic husky kennels. In doing so, we provide empirical justification to the earlier conceptual theorising on nonhumans in stakeholder theory and suggest affective salience that is relevant from a feminist perspective.

The article is organised to first provide a theoretical framework around the human–animal relationships in business, feminist stakeholder theory and feminist care theory. Second, we outline our two empirical contexts and methodological choices. Third, we apply a feminist reading of animal stakeholder attributes in our ethnographic findings through affective embodiment. Finally, we discuss the theoretical and practical implications including avenues for future research.

## Human–Animal Relationships in Business

There is increasing societal and interdisciplinary academic interest in human–animal relationships, including business studies (Connolly & Cullen, 2018; Cunha et al., 2019; Labatut et al., 2016). In a 1995–2015 bibliographical review, 185 peer-reviewed articles featuring animals in business and management were identified (Connolly & Cullen, 2018). However, most described animals in terms of tools, commodities or objects to be exploited by and for humans reflecting a common understanding in business that animals are ours to exploit for profit. This anthropocentric perspective is consistent with the historical evolution of animal domestication which changed the human–animal relationship from one around mutual trust and sharing of resources (in traditional hunter-gatherer societies) to one of human domination and control (where animals are human property) (Ingold, 1994). The animal–industrial complex, where animals are bred and killed in mass numbers to supply the ever-increasing human demand for animal-based protein (see Hamilton & McCabe, 2016), is a stark example of animal exploitation and instrumentalisation. This instrumental framing of the materiality of animals as resources or symbols is also relevant in scholarship; indeed, sociological animal studies suggest shifting towards a realist–materialist approach “to study the animals in the world, not simply the animals in our heads” (York & Longo, 2017, p. 43), thus calling for scholarship to foster more sustainable business activities in practice.

Value judgements in human–animal relationships are also revealed in consumption patterns and animal work (Labatut et al., 2016; Wünderlich et al., 2021). By living in homes and participating in everyday activities, ‘pets’ play an active role in consumption practices and decision-making. Kylkilahti et al. (2016) suggest that humans and their ‘pets’ not only have joint consumption experiences with service providers, but animals also affect decisions and activities beyond ‘pet’ associated consumption, including the type of car to buy, where to work, whom to marry and how to live. Hence, these animals are nonhuman consumers with their own interests and, as a result, can be considered in relation to strategic business practices. Similarly, animal work has gained traction within labour studies that acknowledge, capture and explain the complexities of the work done with, by and for animals (Coulter, 2016). Animal work is common in many organisations, where animals not only work but are regarded in some sense as workers (Coulter, 2016). Considering that millions of humans and animals work side-by-side, animals are not beyond human organising but are rather a constituent part of such organising (Hannah & Robertson, 2017; Sage et al., 2016) and businesses should further consider nonhuman interests in their practices (Wünderlich et al., 2021).

Human–animal ‘work’ relationships have existed for thousands of years, the longest being with dogs used as hunting tools (DeMello, 2012). Consequently, it is logical to explore human–animal relationships at work with dogs. This scholarly “obvious starting point” has already suggested that dogs play functional roles based on their unique canine skills (e.g. in search and rescue roles) or human–canine communication ability (e.g. improving human wellbeing and work climates), in addition to symbolic impacts related to leader values (Cunha et al., 2019, p. 788). Although most animals have not entered work relations freely, there are situations where domesticated animals do voluntary work (e.g. in homes involving care and protection) (Coulter, 2016).

Considering that human–animal relationships are complex based on both ‘love’ and exploitation (Dhont & Hodson, 2020), there is a need to explore the contextual, emotional and affectual relations in practice and their impact on stakeholdership. This endeavour is seen as challenging within the fairness-based stakeholder approach, limited to obligations and constrained to instrumental rationality (Phillips & Reichart, 2000), but feminist stakeholder theory (Burton & Dunn, 1996; Wicks et al., 1994) allows us entry into reconfiguring this challenge. In doing so, we respond to calls to further develop stakeholder theory through a feminist ontology and ethics (Greenwood & Mir, 2019).

## Feminist Stakeholder Theory and Animals

The conceptualisation of feminist stakeholder theory proposed by Wicks et al. (1994) refocuses masked strategies of profit maximisation (with intense competition and power framed as ‘objectivity’) towards strategic solidarity creating value and wellbeing for a network of interconnected stakeholders (see also Spiller et al., 2011). As outlined in the previous section, humans and animals co-create value in many contexts within different types of relationships. A feminist perspective offers the possibility of a shift from a traditional discourse of management focussed on aspects such as hierarchies and control towards one focussed more on collaboration, harmony, open communication and dialogue (Greenwood & Mir, 2019; Ottaviani & Picard, 2019). We suggest taking this further by recognising affectivity as key in relationships, thus informing to whom (and what) value is ascribed. In other words, it is those (and that) we care both for and about that often direct actions and motives in how interests are valued.

How we value animals can be categorised through an ethic-of-care framework, whereby human–animal relationships are either *concrete* (with direct personal human–animal interactions) or *abstract* (with an ‘objective’ distance), ascribing animals *intrinsic* value (animals as self-valued beings) or *instrumental* value (animals as a means to an end, e.g. profit) (Connolly & Cullen, 2018; García-Rosell & Tallberg, 2021). These dimensions form four types of human–animal relationships: ‘no care’ (abstract/instrumental value); ‘care about’ (abstract/intrinsic value); ‘contractual care’ (concrete/instrumental value); and ‘care for’ (concrete/intrinsic value) (Connolly & Cullen, 2018). In terms of ascribing animal stakeholdership, it is worth noting that many exploitative human–animal relationships assume a ‘no care’ or ‘contractual care’ stance due to the foundational instrumentality of the economic relationship. Despite this, the case is more nuanced as there is power in the resistance animals instil through public outcry (often based on both care and justice arguments) and the rise of ‘ag-gag’ laws, both of which are examples of silencing such power by favouring corporate interests over justice and welfare.

Although care and justice are often juxtaposed as elements of feminine versus masculine aspects of morality, in practice these are often intertwined rather than distinct differences of morality (see Held, 2006) and we do not mean to privilege one over the other. But to extend feminist stakeholder theory to include animals, we start with an ethics-of-care stakeholder framing (Burton & Dunn, 1996) to better understand the entangled, interconnected nature of human–animal relationships. Acknowledging current methodological limitations and tensions in fully understanding animal interests (although this relates to human actors too), we invoke a care ethics rising from an embodied perspective (Pullen & Rhodes, 2015). Affective knowledge gained from such a perspective can inform some understanding of animal interests in the hope to promote more responsible business practices.

Ethics of care focuses on affect, compassion and other forms of other-focussed positive feelings as key to moral reflection and may create mutual benefits in stakeholder relational networks if supporting multiple actors in beneficial ways. Acknowledging an entangled, interconnected reality is core to upholding social responsibility as organisational actions affect humans and nonhumans in intricate interwoven networks (Spiller et al., 2011; Waddock, 2011). However, care is often linked to our immediate social structures (e.g. family, friends, colleagues) (cf. Burton & Dunn, 1996), creating a general moral theory which is highly relational in terms of space and time. This relationality and ethics-of-care application of stakeholder theory is also heightened by the notion of ‘social proximity’ affecting other stakeholder attributes (i.e. power, legitimacy and time urgency) (Lähdesmäki et al., 2019). The conceptual article supplied by these authors proposes the notion that the embedded and affective relationships of small business owners in local communities influence the ways in which they make sense of their stakeholder relations and how they prioritise them (Lähdesmäki et al., 2019). Hence, in especially smaller organisations and businesses (which, for example, husky kennels belong to), the emotional and social bonds between actors are important to understand when discussing the stakeholder salience and interests of such contexts. This suggests a need to understand the situationally critical relationships for that specific context or business. Furthermore, when extending stakeholder salience to nonhumans, this social proximity can be seen in the human–animal relationship valuations and how concrete or abstract human–animal relationships are constructed around care (cf. Connolly & Cullen, 2018). However, privileging closer relationships based on social proximity can potentially lead to unethical actions to other vulnerable individuals located at a distance who may suffer as a result of the decision. Thus, Burton and Dunn (1996) suggest adopting a hybrid Rawlsian moral approach in feminist stakeholder theory promoting care as foundational while justice becomes a ‘superstructure’ of morality. This implies that the moral grounding of stakeholder theory requires a broader, political awareness and ‘care’ that goes beyond locality or mere common conventions.

## Ethics of Care for Animals is Political in Perspective and Dialogical in Method

Communication and collective action are stressed in feminist stakeholder relationships (rather than intense competition) (Wicks et al., 1994), but feminist stakeholder theory has not suggested *how* to do this methodologically, beyond human verbalisations, which we believe has stunted nonhuman stakeholder considerations to date. Thus, following feminist animal scholar Josephine Donovan’s (2006, p. 324) sentiments that feminist care ethics “must be political in its perspective and dialogical in its method”, we apply this to advancing animal stakeholdership. As the core political concern is who is considered (i.e. has agency) and how we know their interests (when they lack verbal speech), we draw on the words of disability scholar Sunaura Taylor (2014, p. 110):

a feminist ethic-of-care offers a framework of justice that has the potential to complicate conceptions of dependency (perhaps in a similar vein to disability studies) to understand animals not as dependent beings with no agency, but rather as vital participants and contributors to the world.

The perceptual shift away from victimising and infantilising (some) humans and animals opens avenues to understand stakeholder attributes through a less constrained and conventional manner. In this way, a feminist perspective helps us to move beyond anthropocentric modes of communication to better interpret nonhuman interests. As this assumes a less exploitative relationship based on inherent value rather than pure instrumentality, it requires both humans and animals to move beyond their own species-specific modes of communication, to be more attuned to the ‘other’ and inhabit an intermittent space for collaboration. Such dialogical aspects of communicating with animals have long been suggested in feminist animal studies to include the empathetic noticing of entangled communication, referred to as “emotional qualia” (Donovan, 2014) and “entangled empathy” (Gruen, 2015).

Empathetic noticing is not a “matter of caring for animals as mothers (human and nonhuman) care for their infants as it is one of listening to animals, paying *emotional attention*, taking seriously—caring about—*what they are telling us”* (Donovan, 2006, p. 305, italics added). This includes emotional attention to ‘signs’ in nonverbal communication patterns, such as in facial expressions, body gestures and movements, posture, tone and attention, all of which frequently inform human–animal interactions. Such attention gives rise to a 'quality' of embodied emotions (e.g. felt joy or sympathy), and emotional qualia can therefore help interpret another’s interests. Paying attention to specific interactions and embodied experiences can resist oppression and domination (Pullen & Rhodes, 2015; Valtonen et al., 2020), especially when dealing with marginalised or disadvantaged others. Hence, we suggest that through such ‘listening’, previous critiques of nonhuman stakeholdership become void, as we move beyond limited rationalisations and communication based solely on human verbal speech and agree with Waddock (2011) that verbal speech cannot be a precondition to either stakeholder status or to the ability to hold a dialogue.

These aspects of entangled human–animal communication support a posthumanist ethical agenda and we use these understandings to inform our analytical methods in the hope of an increased multispecies understanding. Hence, we use the concept of affectivity as arising embodied emotional qualia illustrated from our two ethnographic cases. Next, we empirically illustrate our argument of animal stakeholdership, but first briefly introduce the contexts and methodological considerations.

## The Contexts: The Animal Shelter and Husky Kennels

In our empirical illustrations of animal stakeholdership, we draw on ethnographic data from two multispecies organisational contexts: an animal shelter and Nordic husky kennels. Our first context is a not-for-profit animal welfare organisation operating shelters. Each year, millions of unwanted, abused and neglected animals enter animal shelters globally as a way to ‘care for’ animals, but these settings also societally manage them. Our case was a high-intake shelter in a metropolitan city operating according to an ‘open-door’ organisational policy. Referred to as ‘kill-shelters’ due to the animal population being managed according to limited organisational resources by ‘euthanising’^1^ less-desirable animals, this particular shelter cared for over 20 000 animals annually, half of which were dogs. On average, ten animal shelter workers and volunteers attended to the animals on a rotating work roster. The main work tasks of the paid animal shelter workers included daily animal husbandry tasks (feeding, cleaning and medicating the animals), in addition to assessing and ‘processing’ animals according to organisational constraints and policy.

Our second context was for-profit husky kennels in the Nordic tourism industry, where thousands of huskies work, generating millions of euros in annual revenues. Dogs live in outdoor kennels and populations vary from a dozen to 500 dogs per company. There are hundreds of year-round kennels in addition to roaming dog mushers coming to work in the tourist peak season (December–February) from other parts of Europe with their dogs. The majority of sled dogs are Alaskan Huskies (a mix of different northern breeds chosen for their pulling skills) or Siberian Huskies (commonly depicted on marketing material). Husky safaris range from short (0.5–2 km), medium-length (10–40 km) and multi-day rides (2–8 days) and sleds are pulled by four to six dogs (depending on passenger weights). In addition to customer service, workers are responsible for animal husbandry tasks (such as feeding and cleaning the kennels), as well as training the dogs. Huskies start working at 18–22 months old and pull sleds on average for ten years. Their working shifts and resting periods are often planned and once the dogs reach retirement age they work in less demanding tasks (such as being cuddled or photographed by tourists), some are put up for adoption while other companies ‘euthanise’ nonworkers.

## Data Production and Methods

According to Flyvbjerg (2011), case studies are useful in illustrating a nuanced reality and generating deeper understandings about broader phenomena. We utilise two case studies implementing ethnographic methods. In the animal shelter study, 20 semi-structured interviews and 10 months of participant observational fieldwork were conducted in which the researcher worked as an insider in a paid frontline shelter role. This internal researcher positioning allowed for deeper access to the embodied knowledge and experience of the human–animal relationships, as well as building collegial trust for the interviews. Such trust is important as this occupational group suffers work-based moral and emotional conflicts, thus affecting insights gained from other research methods (see Tallberg & Jordan, 2021). Of the interviews, 15 were frontline animal shelter workers (same as the researcher); four held senior management positions and one was an administrative employee. The demographics of the interviewees were 15 women and five men between 20 and 60 years old; the researcher matched the frontline demographic of it being younger women working as animal carers. The interviews were mainly conducted at the shelter, lasting between 30 and 120 min, and were audio-recorded and transcribed. In addition to these, comprehensive researcher diaries were used as data.

The dog-sledding data consisted of semi-structured interviews and social media content generated by tourists and kennels. Based on information gathered through ethnographic fieldwork involving approximately 20 different animal-based tourism companies (reindeer, horses and huskies), a group of 11 companies (which showed a high level of commitment towards animal welfare) were selected for interviews. For the purpose of this article, we limited the data production to five interviews (one included two interviewees) conducted with husky kennel owners. Three of the owners were women while three were men ranging between 35 and 55 years old. The interviews took place on the premises of the husky kennels, lasting 60–90 min, and were audio-recorded and transcribed. The husky data also included user-generated social media content on 27 kennels and eight destination management companies selling husky tours. This comprised 269 publicly available reviews and comments systematically collected between 2016 and 2018 from Facebook, Instagram, TripAdvisor and YouTube.

Both studies were conducted according to national ethical research principles and had gained ethical approval by the respective university ethical boards of the authors. All interview participants were recruited on a voluntary basis and started with a discussion of the research where each interviewee was assured anonymity and the ability to withdraw at any stage.

## Data Analysis

In line with Wästerfors et al. (2014), we engaged in data reanalysis on two ethnographic studies where animals played a central role inside the organisational contexts. By combining these, we focussed on previously underdeveloped themes and theoretical applications of our earlier work (Wästerfors et al. 2014). Together, these form a multi-site ethnography on human–animal work. There is a specific collaborative aspect to our data analysis recognising that “researchers are embodied, socially located humans… like anyone else” (Cornish et al., 2014, pp. 80–81). Considering our studies were based on the long-term immersion of the researchers in the fields, this allowed for a deeper embodied reading of the material with access to insider knowledge. Such insider access may be of particular value in controversial contexts which at times include emotional labour (Tallberg et al. 2014). Thus, the authors followed a collaborative analysis, facilitating a combination of diverse embodied perspectives through distancing and critical reflections (Cornish et al., 2014).

To start, the first author first read all transcripts, diaries and social media comments multiple times and identified 45 animal shelter narratives, 162 husky excerpts and 269 social media comments related to human–animal interactions. The ‘in vivo codes’ of the human–animal relationship were elaborated upon in the author’s own words in a separate column in the analysis (in efforts to create transparency of perspectives and understanding between the authors) (Cornish et al., 2014). Then the first author analysed these texts according to the four human–animal care relationships (Connolly & Cullen, 2018), which is also how the findings are presented. For example, the shelter context revealed a predominant ‘care for’ framing when examining the material between shelter workers and animals. The other authors verified the readings and agreed on the final codes (care for/care about/contractual or no care). The third author analysed elements of affective embodiment in the texts. These were verified by the other authors, discrepancies were once more discussed and the final themes-coding (Boyatzis, 1998) included ‘time’, ‘acts of embodied care’, ‘interspecies communication’ and ‘emotions’. Lastly, the stakeholder attributes (Driscoll & Starik, 2004) were analysed from the texts using an affective embodied reading. As a result, affective salience emerged organically concerning importance in the messy, intertwined and often implicit understandings of the ethnographic data which serve as empirical illustrations of animal stakeholdership.

## Findings: Illustrating Animal Stakeholdership in Two Contexts

This section illustrates animal stakeholdership in three human–animal care relationships: the ‘care for’, ‘contractual care’ and ‘care about’ relationships (Connolly & Cullen, 2018). These categorisations were data-driven and although there existed some ‘no care’ relationships in our data, these were outliers and not included here as these failed to offer any further understanding for animal stakeholdership. Thus, we explore how the stakeholder attributes of power, legitimacy, urgency and proximity (Driscoll & Starik, 2004) are manifested in these relationships through a feminist reading of affective embodiment, hence revealing an alternative, more nuanced understanding of stakeholder salience, as well as animal stakeholdership. Following our understanding of affective embodiment as an entangled process, we categorise our findings according to the stakeholder attributes because in our adopted feminist reading these are not separate entities but overlapping.

## ‘Care For’ Relationships Between *Human Workers* and *Animal Clients*

The mission of the animal shelter was to provide care for animals in need, i.e. to find them an adoptive ‘forever home’ or be ‘euthanised’. While organisational processes were based on a utilitarian ethical framing, reflective of a rationalised, distant relationship of senior leadership to the animals, the ‘care for’ relationship was formed between the frontline shelter workers and the animals. Indeed, these shelter workers’ embodied actions and feelings demonstrated a concrete human–animal relationship in which animal value was intrinsic (core aspects for the ‘care for’ framing). The data were saturated with acts of embodied care (see Pullen & Rhodes, 2015) and interspecies communication (Donovan, 2006) reflected in attempts to ‘read’ the dogs and understand their interests. Such noticing, in combination with the affective moments shared by workers and dogs in difficult physical and psychological circumstances, revealed aspects of worker–dog relationality, collective action and shared solidarity, elements that Burton and Dunn (1996) and Wicks et al. (1994) suggest sit at the centre of feminist stakeholder theory. But this ‘relationality’ moves beyond their conceptualisations as the affective embodiment in the following entangled experience reveals:

I washed Lilly as she closed her eyes enjoying the simple bath. I never wanted to take her back and felt sad at having to put her back into her pen. Wolfgang is getting better, but he’s housed with pretty crazy dogs barking and fence-running. At the end of the day, I went again to sit with him, and he lay in my lap relaxing, even with the yapping cattle dogs behind us running up and down. It was almost meditative for us both in spite of the stress and noise. I took Millie to the exercise yard because she was frantic and she relaxed, playing a bit in the grass away from the concrete ‘jail’. Hopefully she’ll get fostered soon so she can improve and feel better. (Researcher diary)

The embodied care in physically proximate acts of washing, petting, playing and taking the dogs outside along with the emotional connections represent a form of power that differs from the traditional understanding of stakeholder power (such as coercive, utilitarian and normative). It is through these co-constructed, seemingly small events that the animals exert affective power over the workers, giving value and meaning to their work (beyond the event and moment), perhaps as part of their calling to help animals (see Schabram & Maitlis, 2017 for animal shelter callings). Both embodied care and a willingness to listen to the animals create an opportunity to develop close emotional worker–animal bonds. As the excerpt shows, affective proximity leads the worker to side-step the prescribed organisational processes (which included strict time-management protocols) to allow for moments of freedom for and with the dogs, allowing them to express active ‘outer’ freedom in playing and running in a field, while sharing moments of passive ‘inner’ freedom when getting bathed or petted. Within limited organisational and animal-based time urgency, affectivity becomes key to strengthening the interspecies relationships where both human and animal provide attention and care to each other’s wellbeing.

However, affective proximity also led workers to give priority to certain dogs over others, indicative of the care critique (Burton & Dunn, 1996), suggesting the need for a ‘superstructure’ of justice. The excerpt illustrates degrees of privileged care to some ‘special ones’ (who are named and given personhood) while others, those ‘not chosen’, are unnamed and grouped together. Whether this is favouritism or distancing as a pragmatic coping mechanism at not being able to offer the same degree of care to all is a matter of debate. As in other organisational contexts, the human decides who is chosen and given time and close affection, thus upholding anthropocentric and exclusive binaries and hierarchies of who is of value (i.e. those in a concrete, closer relationship) and who is not. But privileging those with whom we have a close relationship is also common in human stakeholder relationships (Burton & Dunn, 1996; Lähdesmäki et al., 2019), suggesting this aspect transcends species barriers.

I think it’s really heart-breaking to see how cruel people can be, seeing an animal come into the shelter and seeing the person behind that animal is directly at fault for that animal’s behaviour and potentially why that animal is going to die that day...[voice breaks and cries]. (Animal shelter worker interview 17)

The caring–killing paradox of animal shelter work was highly salient within most of our ethnographic data, confirming that the workforce suffered stress, burnout and high turnover due to the dissonance between workers’ intrinsic values of being “animal lovers” (and ‘called’ to do the work) versus having to follow instrumental organisational policies of animal ‘management’ (i.e. killing) (see Baran et al., 2012; Tallberg & Jordan, 2021). This paradox suggests a distinct ‘power’ of the animals in highly affecting the workers’ psychological wellbeing whereby negative coping mechanisms, such as substance abuse, were common, but also affecting workers to leave their intrinsically motivated occupations. Thus, with increased dissonance, workers moved away and exited the organisation, while a stronger focus on allowing care caused workers to move towards the care relationships and thus stay. In this context, the power of the affective events (Blackmann & Venn, 2010) is clear: affect moves us (humans) and the animals in this context are stakeholders by highly affecting the workforce’s wellbeing, lifestyle and motivation, as well as the organisational bottom line (by costly turnover).

I don’t see the animals as given enough time or care...this is an unrealistic environment. The animals just need to get themselves sussed out, to settle down, and then they’ll step in the right direction. (Animal shelter worker interview 7)

Here, time was highly tied to care. Although time urgency is a key stakeholder attribute (Mitchell et al., 1997), the tendency to focus on large-scale, immediate effects, such as an oil spill rather than incremental climate change, may hinder the recognition of more subtle stakeholdership (Driscoll & Starik, 2004); this follows the feminist approach of recognising importance beyond the obvious. This was evident by the small spaces carved out with the animals (as our first excerpt illustrates) in the time-poor setting, which builds interspecies work connections based on affectivity beyond the work role. Furthermore, there was a (time) urgency of ‘saving’ lives whereby many workers used their social capital and material bodies (similar to findings by Clarke & Knights, 2019) to foster dogs who would be deemed unadoptable and killed. Much free time was spent fostering and rehabilitating ‘difficult’ animals, or volunteering on weekends by running a public dog hydrobath to bring in more funds. On occasions, workers sacrificed their own wellbeing to help specific animals and it was not uncommon for workers to hide their foster animals from their landlords for more rehabilitation time outside the shelter in a home environment. Hence, the shelter workers used boundary spanning methods that infringed on their personal lives to provide better care for the animals and to save as many as possible through ‘deviant’ measures side-stepping authoritarian organisational protocols. In this way, the animals’ urgent interests were powerful drivers behind how the workers attempted to provide care, beyond the animals’ instrumental organisational value and which we argue provides reasons for animal stakeholdership due to the worker-dog affective salience.

The animals’ affective salience is further illustrated in workers’ memories. These can be important sources informing emotional knowledge as recalling moments have an impact (i.e. power) on the person (see also Haanpää et al. 2020). The following quote highlights how the worker’s memories of affective moments are recollected as co-created, embodied acts, suggesting affectivity is core in sensemaking.

Little moments with the animals you share is the reason I continue to work here...a dog [in emergency care] was starting to get depressed…he was moping the whole way out and I remember when he heard the [owner’s] voice at the end of the hallway...yelped with excitement, ran outside, flicked off the lead and was jumping at his feet and started crying. The dog was crying, I was crying, there was another worker there crying and all four of us, just standing there crying with happiness. I knew then and there why I do what I do. (Animal shelter worker interview 3)

The dog’s happiness and the humans’ responses to this create a shared interspecies affective moment. The humans’ ability to read the dog’s mood and interpret his cognitive states are exemplary of the emotional qualia of ‘feeling with’ the animal (Donovan, 2006). Remembering provides meaning to the worker’s experience in seeing the dog’s life progress positively, suggesting time urgency and proximity as being embodied in the recollection and memory of this shared affective event. Furthermore, the emotional qualia of the situation are powerful in affecting the worker’s identity, giving a sense of meaning and purpose in the work motivated by the dog’s feelings. Power, for both the animals and workers, is based on their mutual emotional ties seen through a lens of *power-with* rather than *power-over*, remnant of Mary Parker Follett’s alternative management views of coactive power (Melé & Rosanas, 2003). This coactive power was further evident in how intertwined the wellbeing of workers and animals were in this context. Here, social and emotional proximate bonds demonstrate how those with closer ties (the workers) attempted to ‘read’ the animals and provide alternative methods to ‘save’ them, while the more distant managers did not. Hence, we argue the shelter animals become legitimate stakeholders through affective, embodied and relational acts as their interests move human workers both emotionally and physically, thus fostering what Pullen and Rhodes (2015) refer to as the “enactment of care and respect for difference as it is lived and experienced” (p.159). In this context, this signifies a desire to care for societally disadvantaged animals and to try and help them, and the high degree of emotional involvement suggests the affective salience of the animals to affect and be affected by actions and care.

## ‘Contractual Care’ Relationships Between *Human Business Owners* and *Animal Workers*

Our husky kennel interviews illustrate a predominant ‘contractual care’ relationship in which acts of embodied care contribute to building instrumentally informed human–animal relationships for the sake of financial success. In spite of the ‘special relationship’ dogs have with (some) humans (Donaldson & Kymlicka, 2011), they are often viewed instrumentally as ‘tools’ in work settings, rather than intrinsically. Acts of care are highly time controlled with strict routines that include different ways of sustaining and monitoring the huskies' living conditions, health, training and rest. Interviews used nonpersonal managerial language reflecting an affinity with masculine, rationalised control (Pullen & Rhodes, 2015) consistent with owning and managing a business. Although managerial reductionism and rationalisations also extend to human workers, their contractual relationship is expressed in employment contracts, while huskies work to be fed and housed. Indeed, the dogs’ lives and bodies were subjugated to rationalities that ensured controlled and efficient work relationships for economic success.

We breed the [animal worker] puppy litters...so they learn the routines...they are often related to each other...they have the same routines, the same rules for everyone. (Kennel interview 2)

Despite the physical proximity in this care relationship, the huskies were seen as a group of workers over which certain sets of conduct generally applied, similarly to human resource management. As such, individual relationships were less common suggesting lower affective proximity than in the ‘care for’ shelter relationship. Instead, strict and equal processes were stressed to ensure fairness among the dogs, indicative of masculine sentiments in the “conceptions of morality as fairness” concerned “with rights and rules” (Gilligan, 1982, p. 19), rather than the care ethics privileging a morality of relationships, including the subjective, situational and emotional (Donovan & Adams, 2007). The highly controlled huskies’ lives included being bred on-site, working in lifetime employment, living in outdoor enclosures (or chained to their kennel), and moving about restrictedly (running chained to each other and the sled). Thus, kennel owners exercised total control over the dogs throughout their lives, making decisions about biological reproduction, as well as, in some kennels, ‘euthanasia’ if the husky became of limited economic value.^2^ Feminist animal scholarship (Gillespie, 2014) has highlighted breeding animals for human desire as a form of sexual reproductive exploitation where animals are subjugated to breed (or not) based on human interests. Many husky kennels used puppies as key tourist attractions for interaction and photo-moments with ‘cute’ puppies. Hence, the dogs were born and designed for work and, from a contractual care relationship, they actively and literally worked for their life.

We try to give equal treatment… but there are specific pack-leaders or dogs who are starved for attention, so when we clean the kennels it keeps begging for pats… they, of course, get more attention from us. (Kennel interview 3)

Although special treatment or differentiated caring for individual dogs was seen as a negative issue by most of the interviewees (stressing policies of equality), the quote illustrates how the dog’s character or pack position sometimes affected this ‘fairness principle’. The instrumental value of a pack leader generated aspects of favouritism, giving her a certain degree of agency and power beyond the ‘lower’ ranked dogs. Hence, organisational rationalisations that exist for human workers extend to animals too. Although there were emotions expressed by husky business owners, some affectivity was reserved for ‘middle management’ dogs (i.e. pack-leaders) suggesting rationalisations of affect based on hierarchical power.

The stakeholder attributes of proximity and urgency were especially present in the need for human workers to be able to ‘read’ the dogs correctly, that is, to understand the dynamics of the dog pack and when to intervene in their group discussions. Such attention to dog communication and sensitivities of listening (Donovan, 2006) follows a heightened sense of awareness in terms of some animal agency and interpreting the interspecies communicative dialogues. In this context, the relational interspecies networks are expressed in the embodied communicative messages, an aspect which is not always cognitive or even conscious but, as Äijälä (2019) notes, in husky sled work humans and dogs need to work intimately together to ensure ‘safe and enjoyable’ tourist rides. The interviews stressed the correct identification and understanding of huskies’ different personalities, stamina and temperament as key in successful tour operations. It was through developing the relationship over time that human workers were able to get to know the dogs, a vital element for optimised matches in sled groupings (to minimise in-group conflicts), and to create higher functioning teams over the dogs’ lifetimes for better tourist experiences.

Caring practices were situationally applied to the dogs’ life stages indicative of constructing differential care practices based on individuals’ needs (Donovan & Adams, 2007). For example, many interviewees discussed different caregiving processes related to the birth and training of puppies, adult training routines, as well as senior retirement or ‘euthanasia’ plans.

[The dogs] retire [when older]. They work full-time for 9-10 years and then we evaluate and lighten their workload slightly. It depends on the dog, some retire already at ten, others at twelve years old still do a few short runs a couple of times a week...it’s good for their mental health to run. Sometimes we use them for the last daily safari, as the pace is much slower. But mostly they teach the puppies how to behave and work. Even if we train the dogs, the best trainers are the senior dogs who role model correct behaviour. We [humans] get off easy. (Kennel interview 2)

Attempts to structure work with the intention of attending to the subjective needs of individual dogs may stem from generating economic value from all dogs, but this can also be a way of noticing and paying attention (Donovan, 2006) to the inherent interests of each dog and their inter-dog relational abilities. Such time urgency was evident in how work and rest was organised, but subscribed to rationalised, bureaucratic processes in the structuring thereof. Still, although many kennels stressed the importance of overall animal welfare, this was highly associated with economic success rather than intrinsic valuations. This may stem from the ‘contractual care’ relationship, although the successful business functioning also benefited the dogs (to get fed and housed). Although similar aspects can be found in the instrumental nature of human work, some businesses give different tasks to the dogs over their lives depending on their abilities and interests, while such tailoring is uncommon for humans involved in manual labour. This may suggest (in some respects) a stronger stakeholder argumentation for these dogs than human workers doing physical labour.

The huskies exert significant power (as a group) over the business functioning and success as tourists come to experience the dogs. Nevertheless, as individuals they exert limited power as they may be exchanged if they do not uphold their contractual duties. Aside from ethical aspects regarding breeding and killing, this context sees the working dogs experience a number of privileges over many family dogs. For example, the puppies are allowed to (often) stay with their families and spend their social lives in packs, running together on a daily basis, at times with regulated ‘free’ time between work shifts. Hence, this specific animal work suggests a higher level of contractual care, as from a political perspective many working animals are denied any downtime, socialisation, stability or continuity in their lives (Donaldson & Kymlicka, 2011, p. 141). But their tightly controlled lives also mean cost-cutting mechanisms as some owners chained the dogs to kennels, justifying acts through commercial arguments (Clarke & Knights, 2019, 2021) and referring to animal welfare reasons (to avoid fighting) in order to save money on dog housing. Thus, in this care relationship, animals are stakeholders in similar ways to human workers in contractual ways to complete the work.

## ‘Care About’ Relationships Between *Animal Worker* and *Human Customer*

There was a predominant ‘care about’ human–animal relationship in the husky social media data revolving around a strong intrinsic valuation of the working dogs and whether they seemed ‘happy’ with their lives. As an ‘abstract’ care relationship, this differed from the two aforementioned ‘concrete’ care relationship framings. Although tourists commented on their memorable and highly affective interaction with the huskies, their experiences were too short to establish close, proximate care relationships. Nevertheless, emotional memories influenced their interpretations of the animal encounters and the embodied reactions resulted in compassion and care (see also Haanpää & García-Rosell, 2020; Pullen & Rhodes, 2015), as well as directing moral action (to post comments). Thus, we suggest that memories of embodied encounters also affect animal stakeholdership in this care relationship. Comments were written reminiscing on husky encounters and allowed tourists to relive the affectivities and emotions of the experiences, thus revealing the affective salience of the dogs to ‘move’ tourists. The relationship developed beyond the confines of the organisation and specific time, suggesting a ‘looser’ coupling of time urgency and proximity in the ‘care about’ relationship. Affective memories created relational networks that directly affected business operations through positive or negative customer feedback. In this way, the dogs were powerful actors in the tourists’ interpretations of the level of care the businesses afforded them. Consequently, the stakeholder attributes of power and legitimacy were highly salient as comments directly impacted business returns. The following comment exemplifies how customers were affectively ‘moved’ to post and speak up for animals:

A husky slave camp…[where] they spend a lifetime in duty to us, being chained, never being allowed to socialise with each other or feel affection...you hear them howl, bark and even cry all day and night long. It’s enough to remind you that they are living beings. (Social media comment on kennel 5)

Tourists buy husky rides due to an interest in experiencing a ‘special’, ‘fun’ animal-nature adventure. Hence, comments predominantly focussed on animal wellbeing with subjective and emotional judgements about the companies’ level of care based on customers’ personal animal experiences. As spokespeople, customers here speak on behalf of the inherent interests of the dogs through their embodied emotions (Pullen & Rhodes, 2015) textually communicated through posts. As Donovan and Adams (2007) explain, animals readily communicate their feelings, ensuring humans are morally obligated to act and limit their suffering when noticing this. Such an obligation is found in the interpretations of the contextual and emotional attributes of the tourist encounters and their embodied understandings of the dogs’ agency. Hence, the dogs’ feelings are interpreted through the subjective customers’ experiences and animal agency can be read differently pending contextual differences, including personal ethical judgements, beliefs and values about animals. Thus, we suggest the stakeholder attributes of power, legitimacy and time urgency are heavily entangled in this relationship, suggesting animal stakeholdership due to emotional and ethical arguments made by their de-facto ‘noticing’ spokespeople (i.e. the tourists) and the responses made due to the felt degree of affective power of the dogs.

Furthermore, comments revealed how wellbeing assessments of the dogs were made in light of those that were treated as ‘family’ and were seen as receiving good care. By posting comments, tourists empathised with the dogs without “letting power [i.e., the businesses with human owners] off the hook” (Donovan & Adams, 2007, p. 3). The for-profit animal tourist context did not mean that the dogs should be denied happiness and wellbeing, according to the tourists. Hence, businesses who were seen to uphold duties to higher moral standings (which tourists saw as ‘family-values’) received more favourable ratings.

Dogs who need more down time get it, while dogs that love to run get sent out more...They treat their dogs like family, and it was really heart-warming to get to see it. (Social media comment on kennel 6)

The material arrangements of the dogs’ housings also produced affective movements and strong reactions among the comments. The emotive and embodied assessments of the level of care affected the reputation and image of the businesses, thus heavily influencing financial outcomes, a clear justification for animals as stakeholders even in traditional stakeholder readings (Freeman et al., 2007). However, we point further to the deep emotional concern as power, thus triggering active care (by writing comments) about suffering and highlighting the hidden harm of instrumentalising others beyond the context in their posts. Adams (2007, p. 221) explains how such “attention to suffering makes us ethically responsible” and denying embodied knowledge (such as one’s entangled empathy about another's suffering) is a way to oppress one’s understanding of the other’s situation. In the comments, interpretations of felt suffering were voiced powerfully, but also advocating for those businesses that were seen as taking good care of the animals. Therefore, emotional urgency and proximity takes place in the immediate situation (between specific individuals) but also affects actors beyond the specific moment through memories and media. We argue that the huskies are stakeholders in this care relationship through the posts relating to the physical, emotional and ethical conditions for the dogs, thus suggesting affective power and salience. In these examples, entangled empathy and a feminist care ethic offers a “relationship…[of] reclaiming the body and its full range of feelings, and reclaiming animals’ bodies” (Adams, 2007, p. 221), therefore giving rise to power and hence agency.

## Discussion: Affective Salience in Care Informing Animal Stakeholdership

This article illustrates how a feminist reading of affective embodiment in human–animal care relationships gives access to new theoretical stakeholder insights. In two contexts, animals are stakeholders in practice through our feminist reading of Driscoll and Starik’s (2004) traditional stakeholder attributes. Inspired by feminist animal scholars (Donovan, 2006; Donovan & Adams, 2007), we illustrate how affectivities in human–animal care relationships affect organisations, thus disrupting the humancentricity and conventionality (Huopalainen, 2020) prevalent within management studies. Hence, we answer calls for de-anthropocentrism in business and management (Connolly & Cullen, 2018; Cunha et al. 2019; Sayers et al. 2019) and develop feminist stakeholder theory (Greenwood & Mir, 2019). To provide ample empirical illustrations of different care relationships giving rise to animal stakeholdership, we have offered in-depth multi-site ethnographic case explorations. Thus, our cases provide deeper knowledge and understanding (Flyvbjerg, 2011) of nonhuman stakeholdership, as well as extending and building upon previous theory (Sandberg & Alvesson, 2020).

We reveal how a feminist focus can shift anthropocentrism in the stakeholder discourse, allowing for a more posthumanist, interconnected understanding of multispecies contexts. The feminist ontological approach allows for knowledge construction and communication beyond human speech and verbalisations as emotions, senses and subjectivity become vital to generating contextual understanding, rather than the rationalised processes often idealised in masculine, simplified and codified contexts (Clarke & Knights, 2019). Through privileging affectivity, the messy, entangled, interdependent multiplicity of “humanimal” contexts (Huopalainen, 2020)—illustrated through the three care relationships in our findings—demonstrate core aspects of animal stakeholdership.

In building our argument for animal stakeholdership, we note that the stakeholder attributes (Driscoll & Starik, 2004) in human–animal care relationships are influenced by embodied emotions, memories and actions giving rise to animals as stakeholders. Hence, we have suggested *affective salience,* based on care ethics, as a new aspect of stakeholder theory and key to understanding stakeholder status beyond earlier rationalised and anthropocentric limitations. Our analysis takes inspiration from Pullen and Rhodes (2015, p. 161) who suggest thinking about organisational ethics from an embodied perspective, as “one that arises from the interaction between people, the embodied effects and affects of that interaction and the indissoluble relation between thinking and feeling”. Even though they refer to people, the embodied perspective allows us, in line with Donovan (2006), to elaborate on the affective qualities between different actors, both human and nonhuman. The bodies of animals and humans possess capacities to affect and be together affected (see Blackman & Venn, 2010; Thanem & Wallenberg, 2015) by communicative patterns and mutual affective interactions. As a result, affective salience is the emotional impact (i.e. power) of the animals to trigger care or actions in humans making animals key actors in ‘care for’, ‘care about’ and ‘contractual care’ human–animal relationships (Connolly & Cullen, 2018).

Attention to the ‘other’ is key in the feminist care approach to animal ethics, and a critical difference to the animal welfare lens frequently adopted in business contexts involving animals, thus offering potential insights into both political and economic systems of oppression (Donovan & Adams, 2007, p. 3) to better understand animal interests. Organisational processes and policies observed across our two case contexts support a rationalised, bureaucratic approach whereby some animals are killed if they are deemed ‘unadoptable’ or ‘unable to work’, thus upholding anthropocentric attitudes on a managerial decision-making level. Nevertheless, those in closer relationships (such as the shelter workers) found executing such policies to be highly problematic, to the extent they faced negative wellbeing and intentions to quit. The animal interests can be felt in the emotional qualia (Donovan, 2006) of the humans in animal care relationships and this qualia is embodied knowledge gained through giving attention, listening and noticing, thus giving rise to agency through embodied events (Haanpää et al. 2020; Pullen & Rhodes, 2015) and actions. While there can be different levels of such understanding (e.g. through differences in reading animal behaviour), it is the empathetic concern and intention behind the affective communication which is of consequence. Thus, stakeholder interests do not necessitate human verbal communicative abilities, opening consideration to a myriad of impactful actors who are affected by and can affect businesses. Dogs are great readers of human behaviour and readily communicate their own needs and wants, but humans often remain ignorant in reading such cues, fixating on human verbal communication, privileging this above all other forms. Traditional (nonfeminist) stakeholder theory falls into this trap of limiting stakeholder status to those who can verbalise their interests, thus restricting the full understanding of potential actors and their impact.

Contractual care is not readily discussed in the human–care discourse, yet when considering the employment relationship a similar instrumental and, at times, exploitative situation may arise. Hence, for this type of care relationship the intrinsic value of the worker (be they human or animal) is not key, rather it is the economic value they bring to the business. But even in this instrumental care relationship, we found aspects of collective action and collaboration to influence the livelihoods of both human and animal workers in entangled ‘interspecies solidarity’ (Coulter, 2016) to produce economic value. Though the human workers are merely laid off in economic downturns, dogs may face ‘euthanasia’. However, in 2020 when international travel to Nordic tourist destinations declined due to the Covid-19 pandemic, some husky kennels tried alternative measures to avoid killing, such as adoptions and sponsorship campaigns. If the dogs were purely seen in instrumental and rationalised ways, as ‘tools’, killing them would have been the most cost-effective strategy at keeping business operations functional. Thus, human–animal emotional and social bonds nuance relationships and weigh powerfully in making such business decisions (to find alternative solutions), illustrating insights into the affective salience of the animals in contractual care relationships to also include a sense of justice and inherent value beyond pure profit. This affective salience, of trying to ‘save’ those in close care, also impacted shelter workers’ wellbeing. Indeed, it is not only the animals who suffer (albeit the ultimate ‘cost’ in death) but also animal carers involved in the shared interspecies dilemma of managing unwanted animals. Hence, affective events and bonds are powerful levers for animal stakeholdership in the instrumental husky contractual care relationship, as well as the more intrinsic shelter relationship.

Animals in business follow wider societal speciesist framings, where some animals are privileged over others based on culturally ingrained human preferences (Joy, 2011; Valtonen et al., 2020) rather than their actual cognitive, social or emotional abilities. This historically complex and often paradoxical human–animal relationship is connected to deeper psychological aspects (Dhont & Hodson, 2020); we often deny the suffering of others to avoid our own pain, creating powerful societal forces that discourage responses to the needs of others or listening to our own feelings (Adams, 2007, p.216). Thus, oppressive behaviours can continue. Clarke and Knights (2021) explain how anthropocentric behaviours and processes in dairy production and consumption make humans “morally indifferent to cruel practices…in consumerist life”. Similarly, in stakeholder discourse, this relates to whose interests are seen to be of value. Although psychological aspects are beyond our scope, they are worth noting in terms of changing public awareness to inspire advances of practical business considerations and theory development. Thus, reflecting on the two normative questions that are foundational to stakeholder theory—What is the purpose of the organisation and to whom does management have an obligation? (Parmar et al., 2010, p. 11)—we illustrate these to include animals in certain contexts, and in such organisations, animals should be seen as stakeholders based on the impact in affective human–animal care relationships which affect the organisation.

Parmar et al. (2010) emphasise the interrelatedness of actors as the basis for management when discussing stakeholdership. In the instrumentally abstract ‘no care’ human–animal relationship (Connolly & Cullen, 2018), animals are purely seen as raw materials, objects or ‘animals-as-food’ (McLoughlin, 2019), rather than sentient beings. Here, conditions for animal stakeholdership are limited due to low emotional and social human–animal bonds. Nevertheless, slaughterhouse research exhibits high emotionality, regulated through negative coping mechanisms (Baran et al., 2016) and highly rationalised framings (Hamilton & McCabe, 2016). Emotional repression and rationalisations in overly exploitative industries are logical as the deeper affective noticing of animal suffering would undoubtedly further question the morality of the industry itself.

What does this mean for stakeholder theory? We believe our findings illustrate the anthropocentric limitation of management studies thus far (Connolly & Cullen, 2018), to fully recognise the impact of animals in certain organisational contexts, thus limiting managerial and strategic understandings of such settings. As stakeholder theory at its core is a moral theory, based on networks and webs of relationships, it should not matter *who* is part of this relationship. Rather, the focus should lie on understanding the situational and contextual elements we suggest are highlighted through affective salience, thus contributing to understanding contexts rather than restricting stakeholdership and agency to (some) humans. Such inclusivity opens up the potentiality for a deeper understanding of multispecies organising towards, hopefully, more responsible business practices for animals and other nonhumans in society.

## Contributions and Concluding Remarks

Considering the predominant anthropocentric ontological line of reasoning in management and organisational studies, it is unsurprising that animals have remained on the periphery of stakeholder theory. Although stakeholder theory represents a way of reconceiving the purpose of the firm beyond the narrow idea of shareholder value maximisation (Freeman et al., 2020), it has continued to reify the managerial discourse focussed on anthropocentric perceptions of organising. Making stakeholder theory more relevant in today’s ethical climate is a timely endeavour and we note recent attempts at using affectivity to explain stakeholder theory in the management classroom (Painter et al., 2020), albeit exclusively for humans. Through a feminist ontology and ethics, we placed relationships at the core of stakeholder theory (Greenwood & Mir, 2019) and developed theoretical and empirical justifications for how to consider animals as stakeholders who are able to communicate their interests and in practice affect (and be affected by) human–animal care relationships. This article makes two key contributions to the stakeholder literature in answering our research questions. First, we contribute to whom should count as a stakeholder in answering the question of *how are animals stakeholders?* Confirming animal stakeholdership is our foremost contribution. Our article is among the first to provide an empirical grounding for the conceptualisation of nonhumans as stakeholders and, to the best of our knowledge, the first article to use a feminist embodied reading to illustrate animal interests in stakeholder theory. Prior studies have offered valuable conceptualisations for nonhuman stakeholdership, but these have remained conceptual and contested. By adopting a feminist ontological stance on how to view stakeholder salience, our study expands upon a different way of understanding agency and thus the impact of nonhumans in multispecies contexts.

Second, we answered the question of *how are stakeholder attributes manifested in human–animal care relationships?* In doing so, we contributed to the theoretical advancement of stakeholder salience highlighting affective salience as a key influencing factor in understanding human–animal care relationships. By drawing attention to the embodied relationality of stakeholders, we develop the earlier conceptualisations of human affective relational proximity (Lähdesmäki et al., 2019) to nonhumans with empirical illustrations. Therefore, we contend that proximity is not only social closeness, but also empathy and care that arise from the embodied effects and affects (Pullen & Rhodes, 2015) in stakeholder interactions. Affective salience allows us to reconsider not only how proximity is understood, but also understanding the stakeholder attributes of power, legitimacy and urgency through an affective lens, becoming a lever of knowledge.

Thus, we theoretically, empirically and to some degree methodologically contribute to develop stakeholder literature extending consideration to animals. This creates an opening for other nonhuman actors, including the natural environment (Driscoll & Starik, 2004) and vital planetary entities (Waddock, 2011). Inspired by the work of feminist animal scholars (Donovan, 2014; Donovan & Adams, 2007; Gruen, 2015; Taylor, 2014), we draw attention to understanding influences and human–animal relationships in a more entangled, embodied manner that resist conventional understandings of agency. This offers new ways to ‘listen’ to marginalised nonhuman and human animals. In doing so, our feminist reinterpretation of stakeholdership goes beyond acknowledging the web of relationships and emotional bonds among stakeholders to highlight the role of nonverbal, embodied knowledge and affectivity in establishing and upholding stakeholder relations. This focus moves away from hegemonic stakeholdership categorisations and ontological understandings of such stakeholder attributes (based on dominance, control and power in making their voices heard in the marketplace) towards an understanding of the affectively charged bonds that shape stakeholder relationships. Such an understanding is needed to advance current recognition of our interdependence with the natural world (Waddock, 2011) in business and society at large. Although our article’s focus has been on animals, our reassessment of stakeholdership implies avenues to explore relationality and affective salience of marginalised humans too, such as those in exploitative industries, modern slavery, elderly health care and refugees.

Our future research suggestions are based on our limitations. First, we focus solely on dogs who are historically intimately connected to humans and as a result there may be challenges to affectively ‘listen’ to more ‘distant’ animal species (see Valtonen et al., 2020) or even nonhuman entities. Thus, we invite future studies to investigate contexts involving a variety of nonhumans to further develop diverse nonhuman stakeholdership understandings. Second, our studies were conducted in Western cultures with certain animal-nature dichotomies and philosophical cultures and so we encourage future nonhuman stakeholder studies from other perspectives, such as indigenous ontological framings. Third, although we draw attention to the need to listen to animals (Donovan & Adams, 2007), we acknowledge our limitations as our data were produced and interpreted solely by humans. Hence, developing further multispecies methods (Haanpää et al. 2020; Hamilton & Taylor, 2017; Tallberg et al. 2014, 2020) and interdisciplinary collaborations with, for example, researchers in cognitive ethology could allow for more inclusive ways to research and develop business ethics (see York & Longo, 2017). Fourth, by focussing exclusively on human–animal relations inside organisations, we paid less attention to understanding care and affective relations between humans and ‘external’ nonhuman agencies, such as the natural environment. Although we recognise that nonhumans have inherent value beyond their care relationship with humans, this article offers a pragmatic step towards the broader recognition of nonhumans and animal rights, even when taking a rationalised business perspective as the animals affect the organisation through their affective relationships. We encourage future studies to develop these aspects further.

Our research not only represents a theoretical advancement of stakeholder theory, but also has wide-ranging practical implications for organisations to reconsider their relations to animals and the nonhuman world. As the Covid-19 pandemic demonstrates, how we treat and value animals affects business and society, forcing new human practices (e.g. social isolation and distancing) as well as wide-ranging economic and social impacts. Hence, opening up to listen and notice what nonhumans are telling us is vital, not only to broaden strategic management understandings but also in how life is managed on this planet towards a more holistic, moral and just manner. Our embodied emotions are good ‘signs’ (Donovan, 2014) to empathetically listen out for. Similarly, we argue that for a ‘new business model’ of the twenty-first century (Freeman et al., 2007), managerial decision-making must consider new forms of knowing and noticing as nonhumans greatly impact our interconnected lives and organisations. This may not only contribute to creating better conditions for animals, but also improve human lives by creating more meaningful work built on shared affective human–animal relationships, thus informing interspecies wellbeing and solidarity towards more humane organisations (Coulter, 2016). Revisiting business theories to include nonhumans is urgently needed as pure humancentric focuses not only limit the possibilities of organisations to address solutions to ecological, health and justice challenges, but create even greater risks for nonhuman and human animals as well as the ecosystem as a whole.

## Footnotes

1 The word ‘euthanasia’, although commonly used in animal-related contexts, refers to *consenting* to ending one’s life. Such consent is not common to seek from animals in shelter contexts (Tallberg & Jordan, 2021).

2 Recently, ‘no-kill’ policies have grown in popularity among some Nordic husky professionals and an animal welfare certification is being designed for the industry.
